# Carbon Nanotubes for Confinement-Induced Energetic Nanomaterials

**DOI:** 10.3390/nano13121845

**Published:** 2023-06-12

**Authors:** Ruben Acevedo, Brigitte Soula, Anne Marie Galibert, Emmanuel Flahaut

**Affiliations:** CIRIMAT, Université Toulouse 3 Paul Sabatier, Toulouse INP, CNRS, Université de Toulouse, 118 Route de Narbonne, 31062 Toulouse Cedex 9, France

**Keywords:** filling, nanohybrids, 1D nanomaterials

## Abstract

Oxidized carbon nanotubes obtained by catalytic chemical vapor deposition were filled with an aqueous solution of nano-energetic materials using a very simple impregnation method. The work compares different energetic materials but focuses especially on an inorganic compound belonging to the Werner complexes, [Co(NH_3_)_6_][NO_3_]_3_. Our results show a large increase in released energy upon heating, which we demonstrate to be related to the confinement of the nano-energetic material either directly by filling of the inner channel of carbon nanotubes or to insertion in the triangular channels between adjacent nanotubes when they form bundles.

## 1. Introduction

In past decades, research and development has focused on the synthesis and characterization of new nano-energetic materials (nEM) for both military (explosives and propellants) and civil applications, such as security systems (circuit breakers and airbags) or energetic microchips able to ignite high explosives. This has highlighted reactive nanohybrids as attractive materials for a wide range of applications, including environmentally clean primers, detonators, improved rocket propellants and explosives, thermal batteries, and many others. The proximity of only a few angstroms between the fuel and oxidizer components within energetic nanomaterials results in a strong acceleration of chemical reaction rate, often described as nano-energetics. The nanostructuration of energetic materials opens real prospects for enhancing both energy and reactivity performance while maintaining or even improving features of control and safety. Nanostructured materials or devices have been explored as additives for the amplification of combustion or to enhance the reaction rate during combustion. Aluminum nanoparticles included in nanothermites [1] were shown to be more efficient than micrometer-scale Al when associated to ammonium perchlorate [2]. Boron nanoparticles stabilized by functionalization have been proposed as an attractive fuel for propellants and explosives [3].

The confinement effect of nEM has been explored. Lower ignition temperatures were observed using “biothermite” (ferritin protein cages loaded with aluminum nanoparticles and ammonium perchlorate or iron oxide) compared to the bulk thermite reaction of micrometer-sized iron oxide particles and Al nanoparticles at 50 wt.% [4]. Energetic nanomaterials incorporating carbon nanotubes (CNT) have also been proposed as an alternative. Carbon materials may play a role in the reaction of various propellants or explosives, lowering the combustion rate and contributing to the reduction in the ignition temperature. In carbon nanotube-supported metal (Pb, Ag, Pd, Ni) or metal oxide (PbO, CuO, Bi_2_O_3_, NiO) catalysts, the synergistic impact of CNT and metal oxide nanoparticles on the decomposition and combustion behavior of energetic materials is responsible for significantly enhanced combustion performance [5,6]. As a preliminary hypothesis, it is assumed that the confinement can take place inside or in between CNT. 

Single-walled carbon nanotubes (SWNT) have been incorporated into thermite mixtures of aluminum nanoparticles and copper oxide nanoparticles, making possible the remote optical ignition of nEM as well as the control of the explosion reactivity [7]. An ignition and an initiation process, further leading to actual detonation, was evidenced for pentaerythritol tetranitrate in contact with SWNT upon irradiation with an ordinary camera flashlight, showing that an efficient heat transfer can occur from ignited nanotubes to an explosive material [8]. Recent investigations on optical ignition of nEM based on MWNT mixed with Al and CuO nanoparticles [9] or ferrocene [10] demonstrated the feasibility of laser [11], Xenon lamp, and LED [10] both in continuous and pulsed modes, resulting in a more complete and uniform combustion. 

Choi and co-workers [11,12] have experimentally verified the existence of accelerated thermal waves with both isolated and aligned arrays of multi-walled carbon nanotubes (MWNT) wrapped in a 7 nm thick cyclotrimethylene trinitramine (TNA, also known as RDX, a largely employed military explosive with activation energy of 0.98 kJ/g [13]) coating. Authors discovered that 22 nm (10 walls) MWNT amplified the reaction velocity by more than 10,000 times and a thermopower wave with a maximal output of 7 kW/kg (around 1.22 kJ/g), whereas for 13 nm (9 walls) MWNT, the enhancement was about 1000 times the original TNA reaction velocity value. Moreover, other classic energetic materials share similar energy outputs as RDX: ammonium perchlorate (AP) has an activation energy of 1.95 kJ/g [14]; HMX (1,3,5,7-Tetranitro-1,3,5,7-tetrazocane) presents a lower energy yield of 0.49 kJ/g [15]; TATB (2,4,6-Trinitrobenzene-1,3,5-triamine), on the other hand, possesses an intermediary value of activation energy—0.81 kJ/g [16].

When the oxidant is actually confined inside the one-dimensional nano-cavity of the CNT, a new class of nEM of higher interest is formed. The agglomeration of the nano-oxidant can be effectively avoided, and in addition, the heat exchange efficiency can be increased due to the good thermal conductivity of the CNT. Highly sensitive copper azide (CuN_3_/Cu(N_3_)_2_) could be in situ synthesized inside a 200 nm diameter CNT, leading to potentially new nano-detonators [17]. MWNT have excellent thermoplasticity and expansibility, which was shown by flowing high-pressure gases from explosive decomposition of picric acid through the multi-wall nanotube channels [18]. CNT with inner diameters around 10–20 nm were filled with potassium nitrate (KNO_3_) by wet chemical method, yielding KNO_3_@CNT nEM, which were integrated with a Cu thin-film microbridge to achieve a micro-electropyrotechnic initiator [19,20]. 

To obtain composite materials of energetic compounds by filling the inner channel of CNT, it is very important to first open them. Therefore, the first step consists of the opening of a CNT by refluxing 24 h at 130 °C in 3M HNO_3_, leading to ox-CNT. Moreover, the concomitant filling of opened double-walled carbon nanotubes by uranyl nitrate has been demonstrated [21], as it was already confirmed for larger MWNT [22]. 

Werner-type cobalt (III) complexes formed by the cation [Co(NH_3_)_6_]^3+^ and one or two other entities with nitrate (or nitrite or perchlorate) ions would be good energetic materials for airbag application, releasing only biocompatible compounds when inflamed (non-toxic metallic oxides and H_2_O, N_2_, O_2_ gases). Among the Werner’s heterometallic complexes previously studied by TDA-TGA to demonstrate their energetic material character [23], Suhard et al. [24] have chosen the Werner’s heterometallic complex [Co(NH_3_)_6_]_2_[Mn(NO_3_)_4_]_3_, which has an initiation temperature of 223 °C, to manufacture a pyrotechnic actuator. Deblitz et al. [25] demonstrated that some new compounds obtained from Werner-type cobalt (III) complexes and azotetrazolate, nitrotetrazolate, picrate, and dipicrylamide ions can be classified as primary explosives according to impact and friction sensitivity studies and combustion studies.

In this paper, we describe the synthesis of a series of new energetic nanomaterials prepared by mixing oxidized carbon nanotubes (oxidized Double-Walled carbon nanotubes (ox-DWNT), multi-walled carbon nanotubes (ox-MWNT) or carbon nanofibers (ox-CNF)) with aqueous solutions of an inorganic compound of the “Werner family”: [Co(NH_3_)_6_][NO_3_]_3_ (labelled “W”). The goal was to explore how confinement of the energetic material either between the carbon nanotubes within a bundle or directly inside the nanotubes may impact the energy release and lead to new nEM. The thermal behavior of these nanohybrids was explored, and the hypothesis of the role played by the confinement is discussed. 

## 2. Experimental Section

Synthesis of carbon nanotubes: DWNT and MWNT were synthesized using Catalytic Chemical Vapor Deposition (CCVD) of a methane and dihydrogen mixture at 1000 °C at the surface of transition metal nanoparticles (cobalt, molybdenum), as previously described in detail [26,27]. The composite powder was obtained after CCVD was processed by an aqueous HCl solution to isolate the carbon nanotubes by dissolution of the catalytic support. These nanotubes were isolated by filtration on a cellulose nitrate filter (0.45 mm pore size) and washed with deionized water until pH neutral. The DWNT sample contained carbon nanotubes with between 1 and 3 walls, with ca. 80% DWNT having inner diameters ranging from 0.5 to 2.5 nm (and outer diameters ranging from 1.2 to 3.2 nm). In the case of the MWNT, the number of walls was between 2 and 7 with inner diameters ranging from 0.5 to 6.5 nm (and outer diameters ranging from 1.5 to 8.5 nm) (Figure S1). 

Carbon nanofibers were purchased from Sigma Aldrich (PR-25-XT-PS ref. 719811). The average inner diameter ranged from 50 nm to 70 nm (average outer diameter ranging from 125 to 150 nm).

Oxidation and opening of CNT (preparation of ox-CNT): Prior to use, all samples were submitted to the same oxidation treatment. In a typical experiment, 200 mg of sample was dispersed in 200 mL of a 3 M nitric acid aqueous solution. The suspension was maintained under reflux at 130 °C with moderate stirring (500 rpm) for 24 h. After filtration and washing with deionized water until pH neutral, the solid sample was soaked in water under sonication for 15 min to ensure a good dispersion and then freeze dried. FTIR analysis was performed, and consistent data compared to previous results were obtained (C=O at 1725 cm^−1^; C=C and C=O at 1585 cm^−1^; OH at 1382 cm^−1^; C-O-O at 1215 cm^−1^, solid state transmission in KBr) [21]. 

Synthesis of the Werner complex [Co(NH_3_)_6_][NO_3_]_3_ (W): This compound was synthesized by oxidation of a cobalt (II) salt in the presence of concentrated ammonia using a protocol adapted from the literature [28]. A total of 16.0 g of ammonium nitrate was added progressively to an aqueous solution of Co(NO_3_)_2_, 6H_2_O (14.6 g in 20 mL). A red solution was obtained, and 0.4 g of activated charcoal and 34.1 mL of a concentrated ammonia solution were added. Cobalt (II) was then oxidized to cobalt (III) by bubbling air through the suspension over a period of 4 h. After filtration and washing by iced water, the solid obtained was suspended at 90 °C in a solution of 260 mL of water and 1 mL of concentrated HNO_3_. The remaining charcoal was removed by filtration and the solution was allowed to precipitate by addition of 40 mL of conc. HNO_3_. The orange microcrystals obtained were washed with ethanol and ethoxyethane and dried in air (reaction yield: 71%). Finally, FTIR characterization in solid state using KBr (vibrations in cm^−1^) was performed: N-H from NH_3_: 3245, 1625, 1325; NO_3_^−^: 1767, 1384, 824; N-Co-N: 326. Elemental analysis found the following quantities: Co, 16.68%; H, 5.27%; N, 36.27%. CoH_18_N_9_O_9_ requires Co, 16.97%; H, 5.24%; N, 36.32%. 

Incubation of the energetic compounds with oxidized nanomaterials: An aqueous solution of the reactive compound (Werner complex (W), picric acid (PA), or ammonium nitrate (AN)) was obtained by adding six droplets of deionized water (ca. 240 mg) to 1.1 mg of the compound. A total of 10 mg of oxidized carbon nanomaterial was then added, and the suspension was bath-sonicated (USC300T, VWR, 45 kHz, 320 W) for 30 s. After 5 h of freezing, the sample was freeze dried for 48 h. For the Werner complex and ox-MWNT, another way of filling was explored: heating the suspension at 80 °C in reflux conditions for 48 h before filtration and freeze drying.

Thermal analysis of the compounds—Thermogravimetric analysis (TGA) and Differential Scanning Calorimetry (DSC): Thermal data were collected on a METTLER TOLEDO TGA/DSC 1 STARe System apparatus under an atmosphere of 20% O_2_ in Ar. A sample of 1 to 2 mg was placed in an aluminum crucible and heated from ambient temperature to 600 °C with a scan speed of 10 °C/min.

## 3. Results and Discussion 

New nanomaterials have been prepared by incubating different oxidized carbon nanotubes (ox-DWNT, ox-MWNT, ox-CNF) with a solution of the Werner complex [Co(NH_3_)_6_](NO_3_)_3_ (W). The resulting nanomaterials are called “W@ox-CNT”. Representative results of differential scattering calorimetry experiments for the Werner complex alone and for the synthesized energetic materials are shown in Table 1 (data of all measured samples are given in Table S1). 

**Figure 1:**
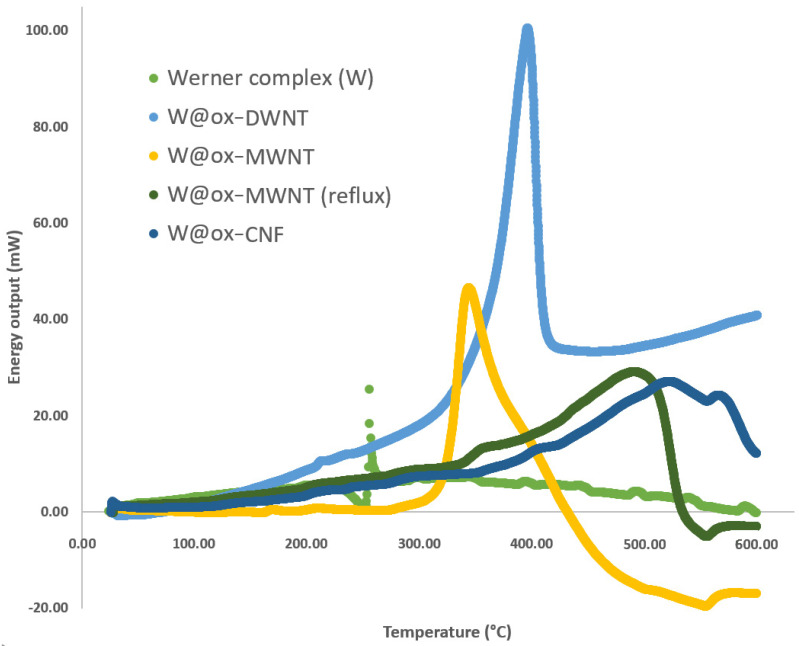
DSC analysis of Werner complex (W), W@ox-DWNT, W@ox-MWNT, W@ox-MWNT (reflux), and W@ox-CNF.

The DSC analysis of W showed a peak at T_w_ = 253 °C, corresponding to E_w_ = 0.5 kJ/g of released energy (Figure 1 and Table 1). This complex is a good candidate for the preparation of nano-energetic materials (nEM) since the combustion occurring at 253 °C was brief, giving a very narrow peak in the DSC graph (full width at the half maximum—FWHM—of 3 °C). When this complex was included in carbon nanomaterials, the amount of energy was significantly enhanced: 14.6 kJ/g with W@ox-DWNT, 23.3 kJ/g with W@ox-MWNT, and 12.1 kJ/g with W@ox-CNF (Figure 1 and Table 1). The ΔE values (ΔE = E − E_w_) correspond to the increase in the released energy due to the confinement of the complex inside the different nanocarbons. The factor or enhancement of the released energy (E/Ew) in filled CNT samples was up to 56. In Table 1 are also shown the values of the maximum temperature of DSC peak obtained for each type of sample (Max. Peak T), and this temperature may be considered the temperature of combustion of the sample. Another effect of the confinement inside carbon nanomaterials is the enhancement of this temperature of combustion for all samples, and up to 515 °C for the W@ox-CNF sample.

Samples prepared in reflux condition to ensure the filling of the ox-MWNT presented a much higher temperature of combustion than the samples filled at room temperature. From Table 1, we can see that the amount of released energy obtained with W@ox-MWNT was ca. 28 kJ/g, when this value was from 12 to 15 kJ/g with the other samples. This may be related with the values of the inner diameter of these different nanotubes: for the multi-walled tubes, the inner diameter values ranged from 0.5 to 6.5 nm, with 98% of these values greater than 1 nm (Figure S1), which may correspond to the steric hindrance of the ionic complex W (0.6 nm for the octahedral metallic cation and 0.27 nm for the nitrate ions); in DWNT, the inner cavity diameters are smaller (0.5 to 2.5 nm) and 40% of the values are less than or equal to 1 nm, thus only a portion of the DWNT were suitable for filling by the Werner complex [27]. For the CNFs, a low filling rate may be explained by a much larger internal diameter (50 to 70 nm), possibly allowing the release of the Werner complex during the drying step. The values of released energy observed with W@ox-MWNT, which are 1.5 to 2 times higher than with other samples, may correspond to an optimal situation of containment of the complex. It is likely that the containment may modify the complex geometry by modification of the bond lengths and modify its activation energy. These kinds of modifications of bond length were already evidenced in the case of the filling of the same kind of CNT with inorganic compounds such as KI [29], PbI_2_ [30], or even tungsten polyoxometalate [31]. Moreover, the FWHM was also affected by the confinement: the peaks were larger in filled nanocarbons with a factor of enhancement going up to 40, from 3.2 °C in W to 52.0 °C in W@ox-DWNT and up to 128.0 °C in W@ox-CNF. 

For comparison, two classical energetic materials were also tested: ox-DWNT were incubated in a solution of picric acid (PA) or ammonium nitrate (AN), using the same procedure as for the Werner complex (Table 2). The same phenomenon was observed: the amount of energy increased from 11.2 kJ/g for picric acid to 15.0 kJ/g for the ox-DWNT impregnated by PA, and from 2.2 kJ/g for AN to 14.7 kJ/g for the ox-DWNT impregnated by ammonium nitrate. It should be noted that even if the energy obtained from these three EM used alone are of different ranges, the corresponding nEM lead to almost the same value, around 15 kJ/g. It is important to note that the compound W@ox-DWNT has an advantage compared to the homologous compounds with PA and AN: the energy of 15 kJ/g is released at a temperature of 396 °C for W@ox-DWNT, while it is necessary to reach 566 °C and 586 °C for PA@ox-DWNT and AN@ ox-DWNT respectively to obtain the same liberated energy value.

Steric hindrances of the selected compounds picric acid [35] and ammonium nitrate [36] of the order of 0.7 nm are similar to that of the Werner complex: [Co(NH_3_)_6_]^3+^, octahedron inscribed in a sphere of diameter of 0.6 nm [37]. As the amount of sample used in the experiments is the same for the three nEM, it may be concluded that a containment effect is responsible for this increase in the released energy. The theoretical maximum heat release was calculated as the sum of the values obtained for the individual components. In the case of W@ox-DWNT and AN@ox-DWNT, the experimental value of the normalized energy output is higher than the sum of its components, demonstrating a clear synergistic effect. However, in the case of PA@ox-DWNT, no increase was evidenced. This raises the question of a possible different mechanism of interaction between the nanotubes and the energetic material in this case. The fact that PA may be more likely to be adsorbed at the surface of the bundles of carbon nanotubes instead of getting either inside or between the nanotubes may be one possibility. 

As observed for the W@ox-DWNT sample, the temperature of combustion was higher when picric acid or ammonium nitrate were inserted in ox-DWNT: from 300 °C when PA [38] or AN [39] were used alone to 566 °C in PA@ox-DWNT and 586 °C in AN@ox-DWNT. Therefore, in every case, the containment inside the nanocarbons leads to the stabilization of the nEM and therefore to a higher temperature of combustion. 

**Figure 2:**
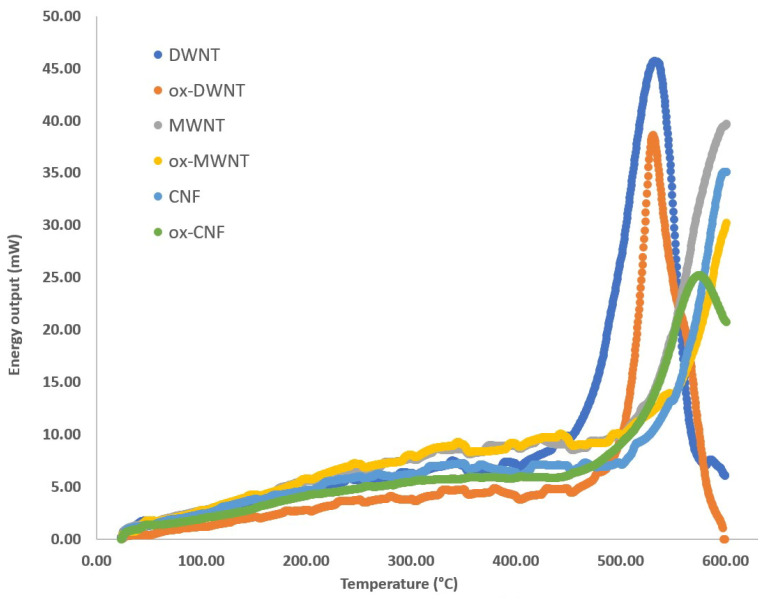
DSC analysis for each carbon nanomaterial and its oxidized form (Al crucibles limited to reach higher temperatures than 600 °C).

Since the values of energy obtained are almost the same whatever the nEM contained in DWNT, is it possible that this energy only corresponds to the DWNT combustion? To answer this question, the different nanocarbons used (CNT and ox-CNT) have been studied by DSC, and the curves obtained are shown in Figure 2.

The quantities of energy released by different nanocarbons samples (DWNT, MWNT, CNF) and for the corresponding oxidized nanocarbons samples (ox-DWNT, ox-MWNT, ox-CNF) are always lower than when they are filled with a nEM, suggesting a catalytic effect of the nEM. This effect is also visible on the combustion temperature. The combustion of carbon materials alone begins between 500 °C and 550 °C and occurs after the limit temperature of the experiment, namely 600 °C, except for the DWNT and the ox-DWNT, for which the released energies of 11.9 kJ/g and 6.7 kJ/g are observed at the temperatures of 533.0 °C and 530.7 °C, respectively. Therefore, the filling of nanocarbons with a nEM can explain the amount of released energy.

Despite the lower energy release observed for ox-CNT, the presence of oxygen functions is an advantage for the design of nEM, along with the fact that oxidation treatments lead to cutting and opening of the CNT [21]. This opening ensures the filling of the nanocarbons, and it may be the key point for the energy output. The rate of opening (and consequently the rate of filling) may differ for each type of CNT.

**Figure 3:**
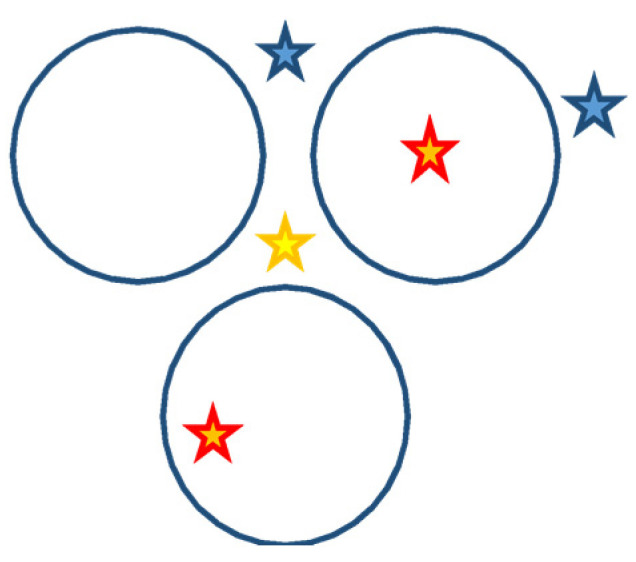
Schematic view of the smallest bundle composed of three CNT (blue circles), with the locations of different adsorption sites (blue, red, and yellow stars).

The DSC data showed that the released energy is indeed related to the filling of the various tubes and not to a simple adsorption of the complex at the surface since in all cases we obtained a single peak of energy instead of two, as we may expect for an adsorbed compound (distinct combustions of the metallic complex and of carbon). The carbon nanotubes stabilized the complex until they were both burnt. This stabilization of the complex may occur inside the nanotubes but also within the nanotubes in a bundle, where sites with a similar size are present. Figure 3 shows five different possible locations where a substance may be found in/out of a CNT bundle. The first two are within the nanotubes: either absorbed onto the inner wall or cantered in the channel (red stars). The third corresponds to the insertion between the nanotubes forming the bundle (yellow star). The last ones (blue stars) are located at the surface of the nanotubes, either in a groove between two adjacent nanotubes, or not.

Unfortunately, such small inorganic complexes are too small and do not have enough electronic density to be evidenced even by HRTEM. Filling was evidenced in our previous work [21], in which we demonstrated that oxidized DWNT were readily filled by a solution of uranyl nitrate in similar conditions.

Compared to classic energetic materials such as AP, HMX, RDX, and TATB, which are in the energy output range of 0.5–1.9 kJ/g, nano-energetic W materials based on oxidized DWNT and MWNT present an improved performance: 14.6 and 23.3, respectively (energy is augmented between 12 to 30 times). The reaction intensity is significantly higher for the nanohybrid subjects of this work, resulting in thinner peaks of DSC. This behavior can be explained by both the confinement effect and the distribution of the energetic material in the available sites (Figure 3).

Few values were found concerning the amount of energy obtained by decomposition of energetic materials, including nanocarbon. DSC studies on mixtures of powdered ammonium nitrate with activated carbon showed that the presence of 4 to 10% of carbon allowed to increase the heat release from 0.8 to 3.4 kJ/g at 180 °C, these values remaining much lower than in this work, due to the absence of confinement effect [40]. Similar low energy values were obtained with a mixture of nano-sized nitrocellulose powder and 1% of MWCNT (1.9 kJ/g at 188 °C) or with a mixture of CNT and nanosized iron oxide (1.8 kJ/g at 186 °C) but with a 20% burning rate increasing in this last case [41]. The thermal decomposition of a composite of MWCNT grown at the surface of Al_2_O_3_ particles was found to be exothermic with a maximal energy value of 6.8 kJ/g at 556.5 °C [42]. Scarce values of DSC studies were found corresponding to the decomposition of energetic materials embedded in carbon nanotubes: the DSC study on KNO_3_@MWCNT samples showed a reaction enthalpy of 0.9 kJ/g at 387 °C [19]. The comparison of our results with the literature thus supports the hypothesis of the importance of filling (or at least confinement) to achieve high-efficiency nano-energetic materials.

## 4. Conclusions

Carbon nanotubes present unique properties, among which the wave propagation modes and confinement effect are of special interest here. Our results show that the energy output of energetic compounds is especially enhanced when they are confined within CNT or possibly also in the space between nanotubes in bundles. In the case of the Werner complex, this work evidenced an increase in thermal energy from 0.5 kJ/g for the complex alone up to 23.3 kJ/g for the same material inside oxidized MWNT, and a lower yield of 14.6 kJ/g for oxidized DWNT. Further work will be focused on other energetic nanomaterials such as nanothermites (AlMo(VI) oxide and aluminum-based nanoparticles) and the analysis of ignition mechanism as well as propagation of heatwaves by numerical simulations. Although this work is still rather preliminary, the high-energy output in a narrow temperature range looks promising for applications where a high velocity is required, such as airbag safety devices for example. 

## Figures and Tables

**Table 1:** DSC representative data for each type of nanomaterial with the Werner complex.

Compound	Normalized Energy Output (kJ/g)	ΔE = E − E_w_ (kJ/g)	E/E_W_	Max. Peak T (°C)	Δ T = T − T_w_ (°C)	FWHM (°C)
Werner complex (W)	0.5	0	1.0	253.2	0.0	3.2
W@ox-DWNT	14.6	14.1	29.0	396.3	143.1	52.0
W@ox-MWNT	23.3	22.8	47.0	342.2	89.0	59.5
W@ox-MWNT (reflux)	28.1	27.6	56.2	504.8	251.6	113.3
W@ox-CNF	12.1	11.6	24.0	515.3	262.1	128.0

**Table 2:** DSC values for nEM, ox-DWNT, and nEM@ox-DWNT (nEM = W (Werner complex), PA (picric acid), AN (ammonium nitrate)). Calculated normalized energy output values are indicated into brackets and correspond to the sum of the values of each component.

Compound	Normalized Energy Output (kJ/g)	Maximal Peak Temperature (°C)	FWHM (°C)	
W	0.5	253.2	3.2	This work
PA	11.2	300		[32]
AN	2.2	293.0		[33,34]
ox-DWNT	6.7	530.7 (should. 546)	31.0	This work
W@ox-DWNT	14.6 (7.2)	396.3	52.0	This work
PA@ox-DWNT	15.0 (17.9)	566.0	30.0	This work
AN@ox-DWNT	14.7 (8.9)	585.6	38.0	This work

## Data Availability

Experimental data are available on request.

## Supplementary Materials

The following supporting information can be downloaded at: https://www.mdpi.com/article/10.3390/nano13121845/s1, Figure S1: Distribution of the number of walls and diameters (inner, outer) for the MWNTs used in this work; Table S1: DSC representative data for each type of nanomaterial with the Werner complex.
