# Supplementary material for: Carbon Nanotubes for Confinement-Induced Energetic Nanomaterials
Source: Nanomaterials (Basel). 2023 Jun 12;13(12):1845. doi: 10.3390/nano13121845 (PMC10304134; doi:10.3390/nano13121845)
Supplement: Supplementary file 1 [file nanomaterials-13-01845-s001.zip › nanomaterials-2390775-supplementary.pdf]

**Carbon nanotubes for confinement-induced energetic nanomaterials**

*Ruben Acevedo*, *Brigitte Soula*, *Anne Marie Galibert*, and *Emmanuel Flahaut**

CIRIMAT, Université Toulouse 3 Paul Sabatier, Toulouse INP, CNRS, Université de Toulouse, 118 Route de Narbonne, 31062 Toulouse Cedex 9 - France
E-mail: emmanuel.flahaut@univ-tlse3.fr

## Supplementary Data

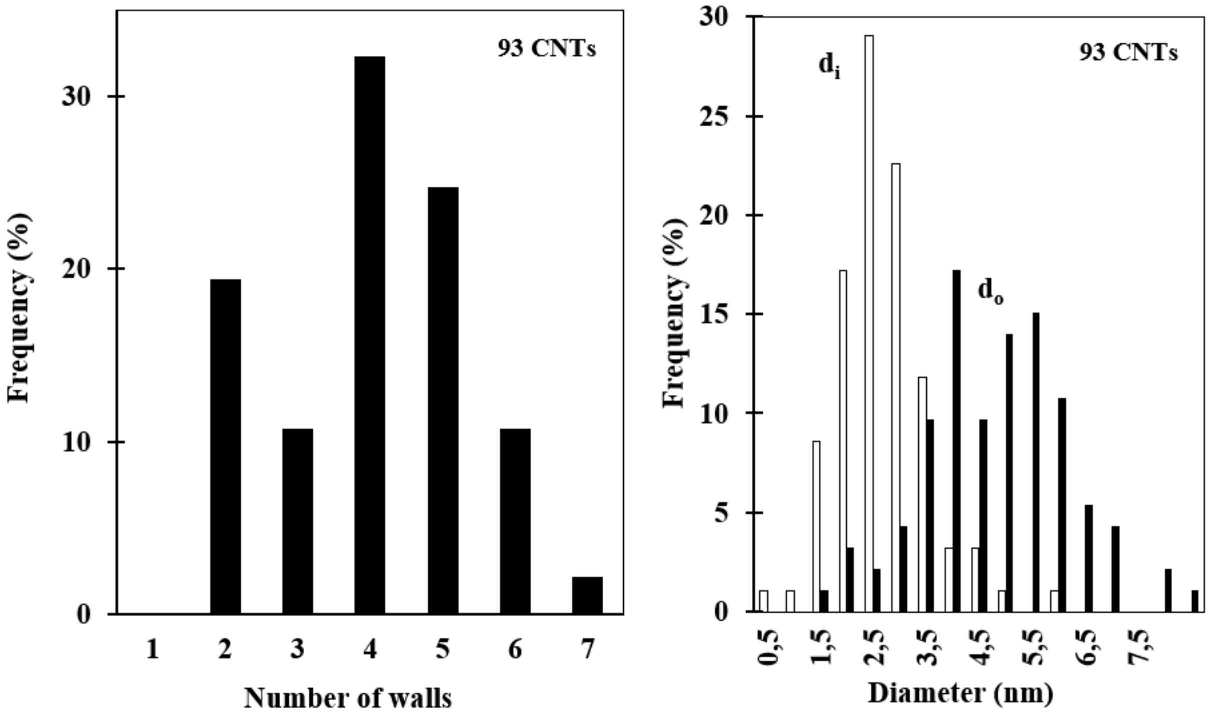


**Figure S1.** Distribution of the number of walls and diameters (inner, outer) for the MWNTs used in this work.

**Table S1.** DSC representative data for each type of nanomaterial with the Werner complex.

**The compounds and their data in bold are those listed in table 1 of the publication.**

| Compound | sample name | mass (mg) | Normalized energy output (kJ/g) | Max. Peak T (°C) | FWHM (°C) |
|---|---|---|---|---|---|
| Werner complex (W) | **AGA305** | **1.02** | **0.5** | **253.2** | **3.2** |
| | commercial | 1.04 | 0.33 | 251.7 | 3.4 |
| W@ox-DWNT | **RA070314_ImW** | **1.0** | **14.6** | **396.3** | **52.0** |
| W@ox-MWNT | AT2+MW | 1.88 | 21.41 | 350.8 | 68.0 |
| | AT5+MW | 1.28 | 14.62 | 470.0 | 65.0 |
| | AT6A+MW | 1.1 | 21.86 | 372.5 | 93.0 |
| | **AT7+MW** | **0.92** | **23.3** | **342.2** | **59.5** |
| | CCIAL+MW | 0.82 | 26.79 | 380.0 | 100.7 |
| W@ox-MWNT (reflux) | RAT8E+MW | 1.68 | 22.65 | 490.0 | 90.0 |
| | **RAT8L+MW** | **0.85** | **28.09** | **504.8** | **113.3** |
| W@ox-CNF | AT2+NF | 1.03 | 15.06 | 512.8 | 121.7 |
| | AT5+NF | 1.08 | 13.21 | 519.8 | 84.3 |
| | AT5'+NF | 1.47 | 12.0 | 538.8 | 92.4 |
| | **AT6A+NF** | **1.03** | **12.07** | **515.3** | **128.0** |
| | CCIAL+NF | 0.62 | 12.18 | 515.5 | 86.9 |

Thermogravimetric analysis data for the samples with names in bold in table S1 are shown in the next pages.

# AGA305

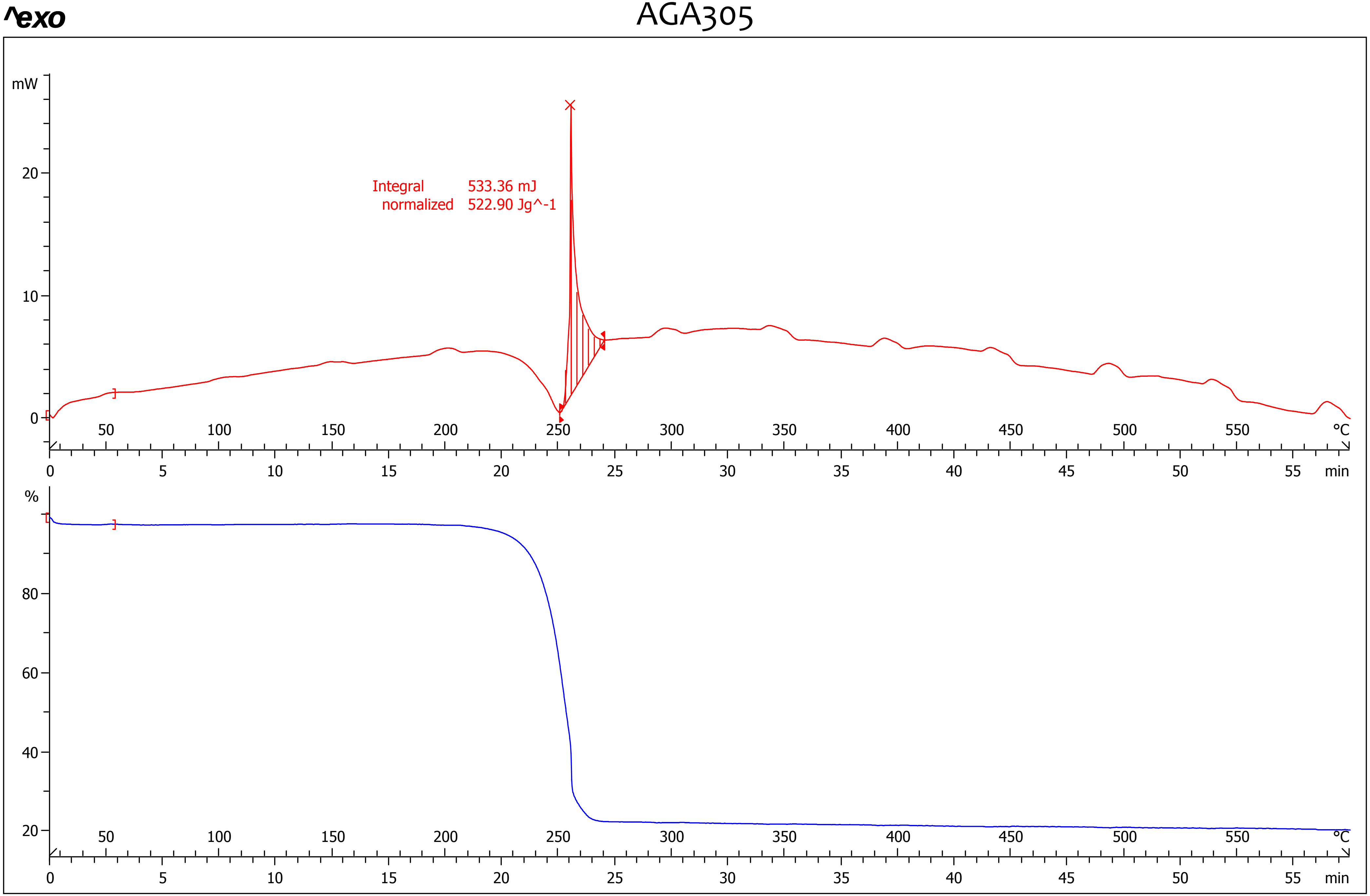


CIRIMAT: METTLER

STAR^e SW 10.00

^exo **140319-RA070314-Im-W-DW** **19.03.2014 13:16:13**

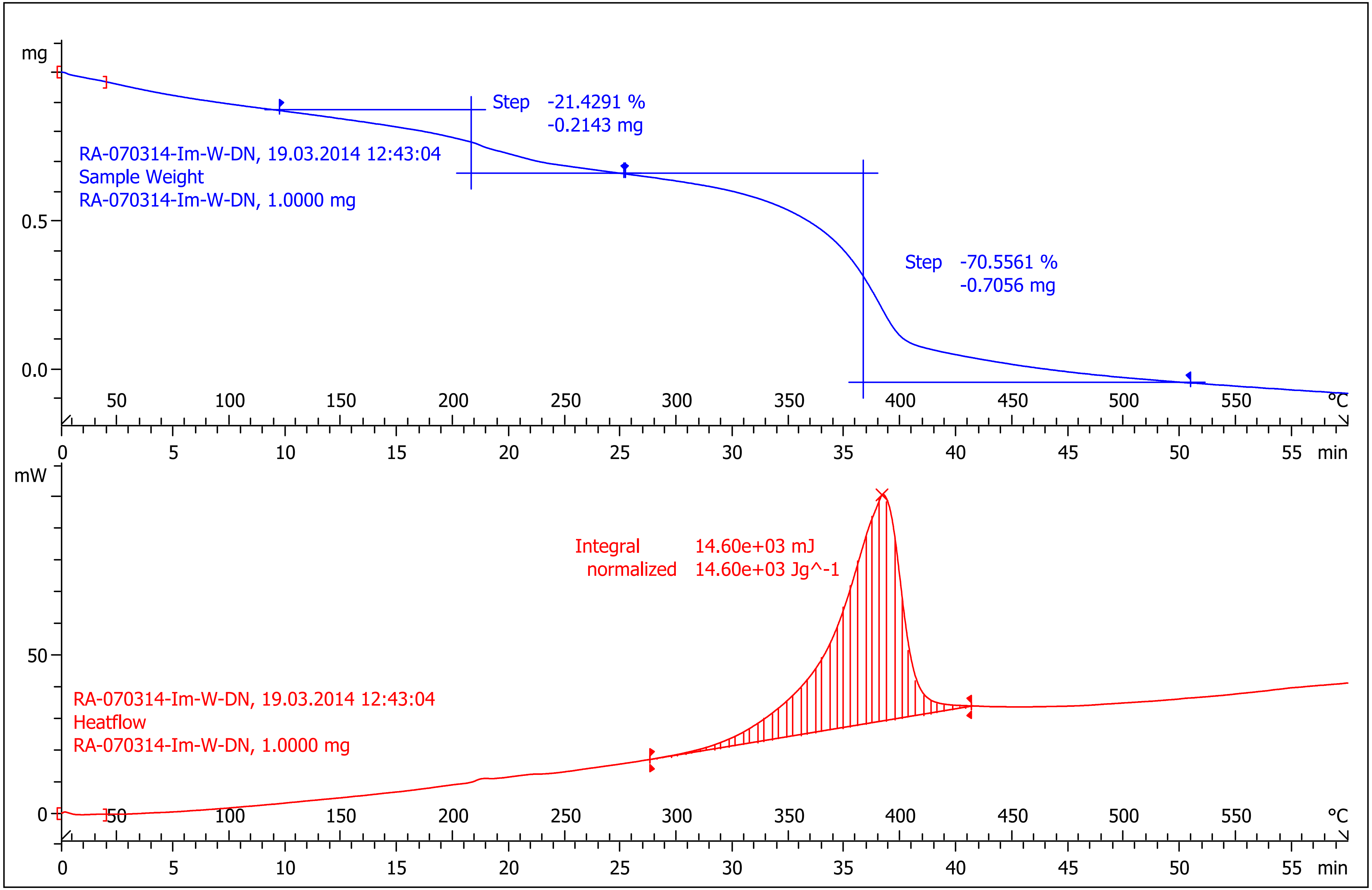


**CIRIMAT: METTLER** **STAR$^e$ SW 10.00**

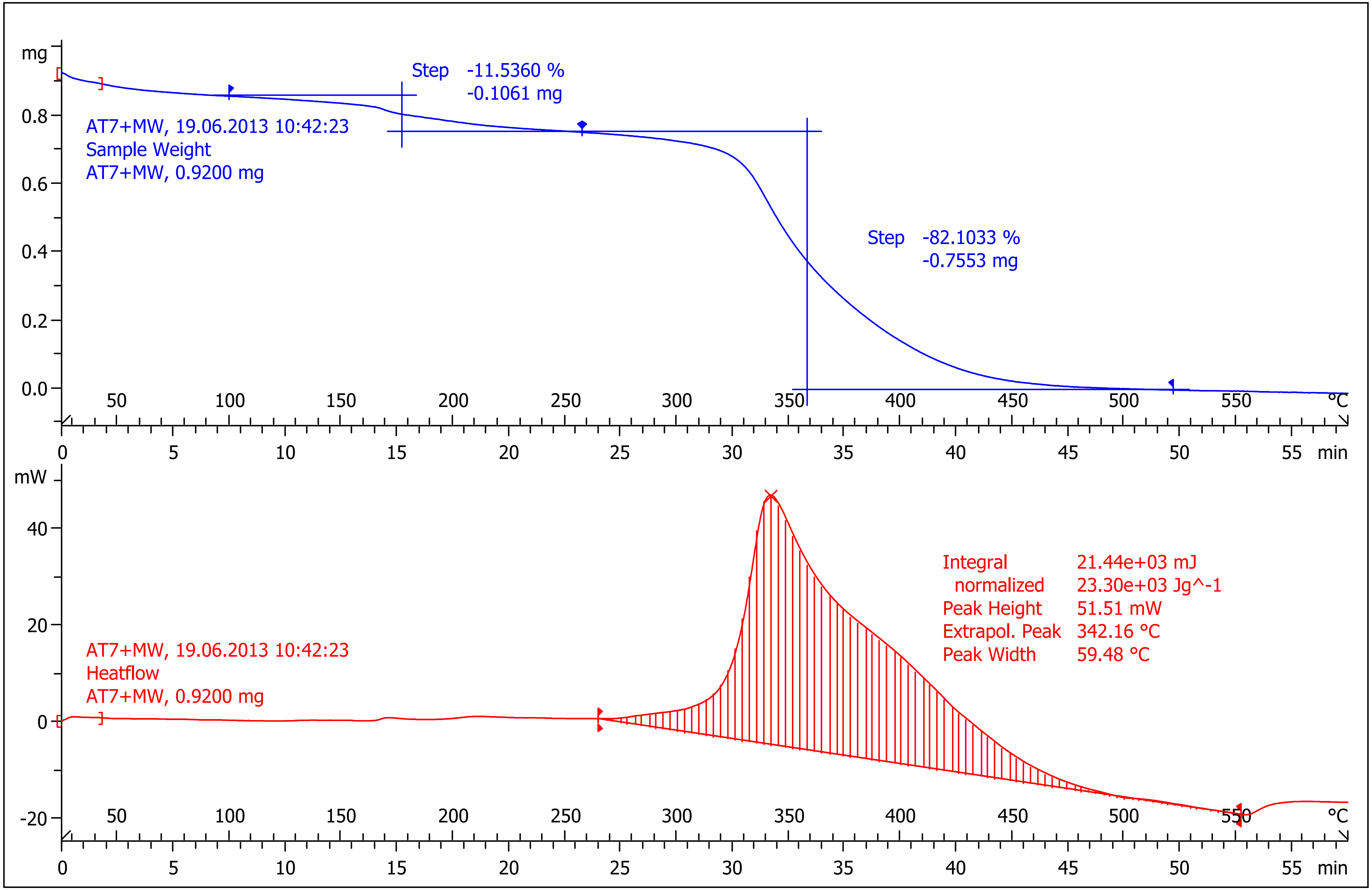
mg
Step -11.5360 %
-0.1061 mg
AT7+MW, 19.06.2013 10:42:23
Sample Weight
AT7+MW, 0.9200 mg
Step -82.1033 %
-0.7553 mg
0.8
0.6
0.4
0.2
0.0
50 100 150 200 250 300 350 400 450 500 550 °C
0 5 10 15 20 25 30 35 40 45 50 55 min
mW
40
20
0
-20
Integral 21.44e+03 mJ
normalized 23.30e+03 Jg^-1
Peak Height 51.51 mW
Extrapol. Peak 342.16 °C
Peak Width 59.48 °C
AT7+MW, 19.06.2013 10:42:23
Heatflow
AT7+MW, 0.9200 mg
50 100 150 200 250 300 350 400 450 500 550 °C
0 5 10 15 20 25 30 35 40 45 50 55 min

^exo RAT-8L 17.06.2013 10:02:27

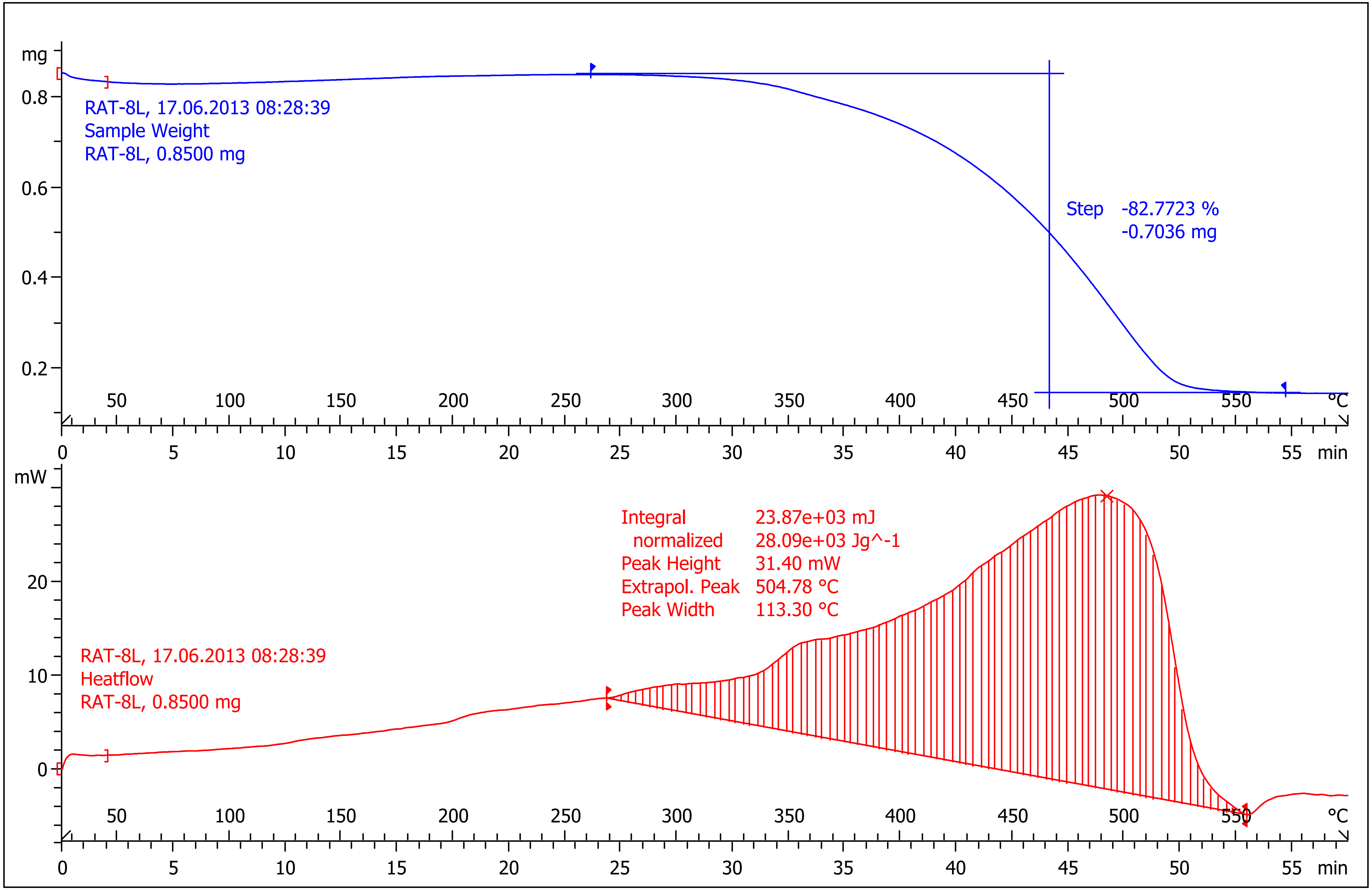


CIRIMAT: METTLER STARe SW 10.00

^exo 130604-AT6+NF 05.06.2013 07:12:31

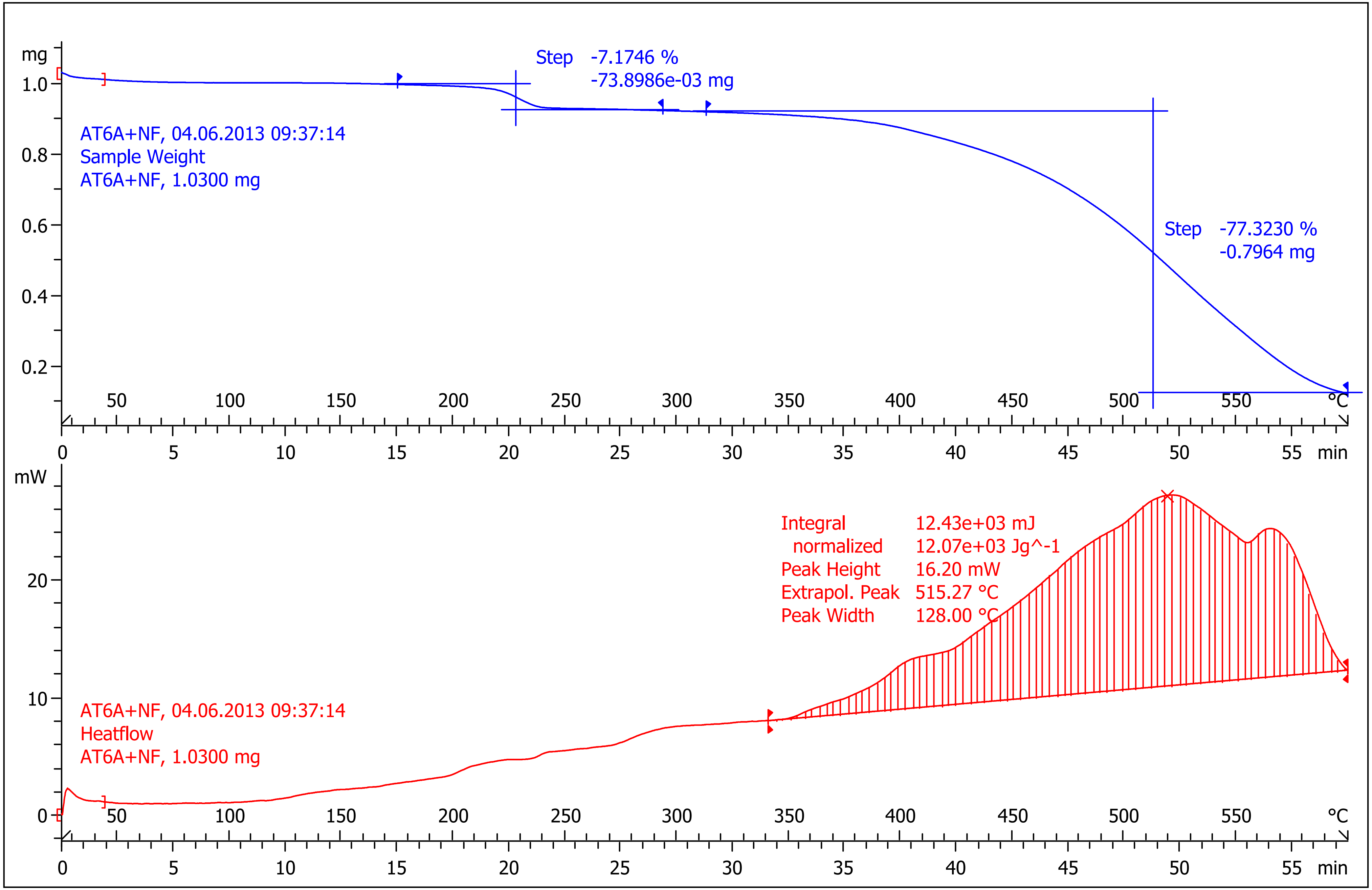


CIRIMAT: METTLER STAR^e SW 10.00
